# Supplementary figures and images for: Brettanomyces bruxellensis wine isolates show high geographical dispersal and long persistence in cellars
Source: PLoS One. 2019 Dec 18;14(12):e0222749. doi: 10.1371/journal.pone.0222749 (PMC6919574; doi:10.1371/journal.pone.0222749)

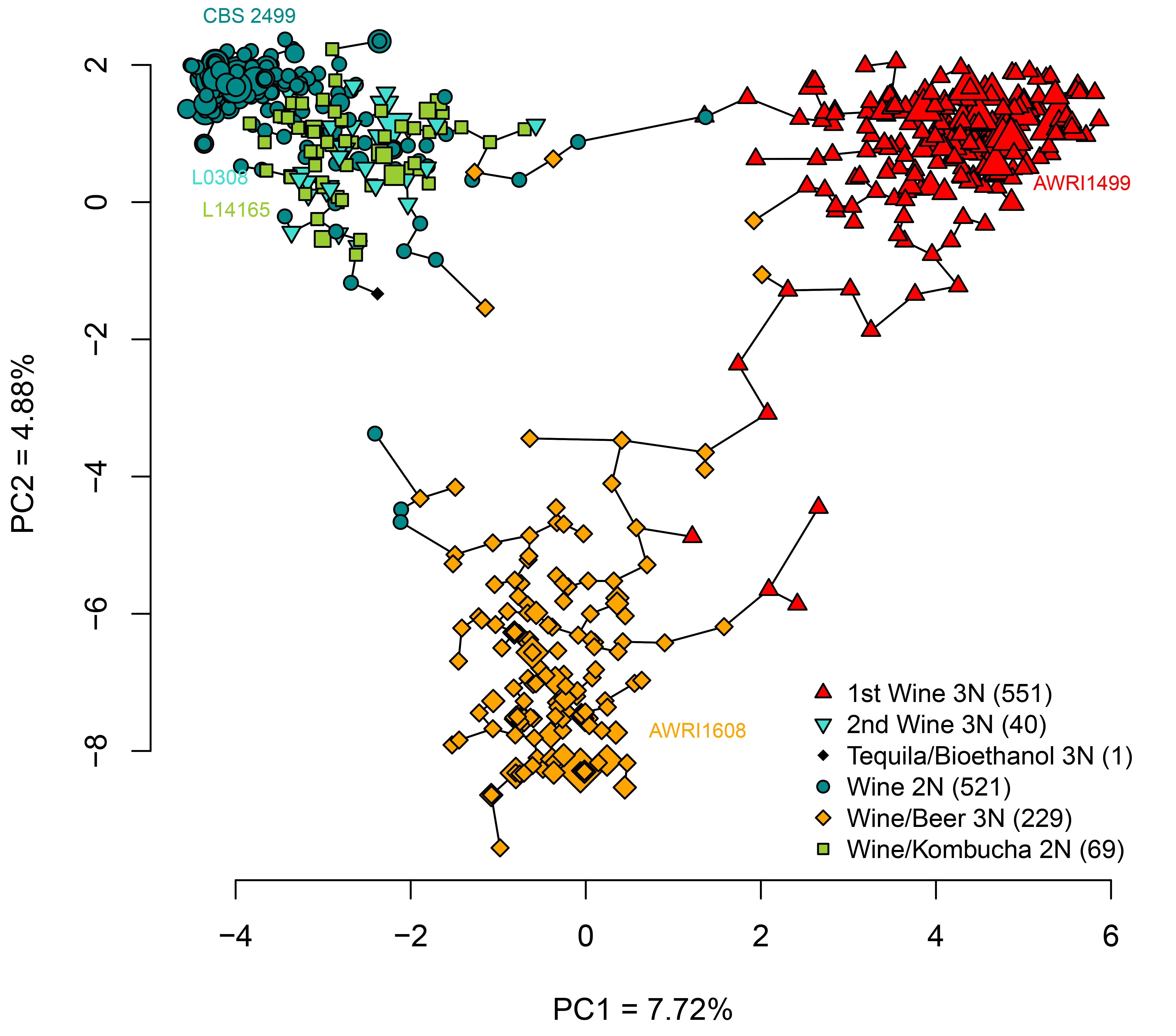

Supplement: S1 Fig — 1411 strains were genotyped using 12 microsatellite markers. For each strain (and each locus) showing 3 alleles, one of the three alleles was randomly removed to produce a randomly 2N-constrained dataset. A PCA was then performed using the R ade4 package. Only the two first axes (principal component, PC1 and PC2) were represented. The connection network and minimum spanning tree was built using the chooseCN function from R adegenet package. For genetically identical isolates (aka ‘clones’), the size of the points is log10 proportional to the number of isolates. (TIF) [file pone.0222749.s002.tif]
